# Case of Fracture of Skull, with Loss of Cerebral Substance

**Published:** 1845-10-01

**Authors:** Alden S. Sprague


### Case of Fracture of the Skull, with loss of Cerebral Substance.

Communicated for the Buffalo Medical Journal, by ALDEN S. SPRAGUE, M. D.

I was requested June 25th 1845, by Dr. Harris, of this city, to visit John Shea, a patient of his living in Lancaster, 8 miles from the city, who had received a severe injury of the head. From himself and friends I obtained the following history of the case. He left the house on the day previous, for the purpose of chopping in the woods. He recollected being at work at 3 P. M. Between the hours of 5 and 6 P. M. he came home severely wounded in the head. A large piece of wood was taken from the wound with much difficulty, and a messenger dispatched for Dr. Harris. Dr. H. arrived during the night, and finding a severe injury, on his return to the city, desired me to visit the patient with him. We saw the patient together at 3 P. M., nearly 24 hours after the accident. He was then perfectly sensible, and free from pain; pulse about 80; skin cool. The wound was situated on the right side of the head, at the junction of the frontal and parietal bones, superiorly. The scalp was shaved, and the wound cleansed of blood and brains, and also numerous small pieces of bone. On further examination, a portion of the inner table 1¼ inches long and ¾ of an inch broad, was found to be crowded into the substance of the brain to the depth of 2¼ inches. This was readily raised by an elevator and forceps, the wound thoroughly examined, and several small pieces of bone removed, a quantity of the substance of the brain necessarily escaping. After having satisfied myself that the wound was free from all extraneous substances, pledgets of lint, and bandages were applied, with injunctions to keep the patient perfectly quiet, and to allow nothing but cold water until he could be again visited. The subsequent treatment is detailed in the subjoined note from Dr. Harris:

Dr. Sprague: Buffalo, August, 1845.

Dear Sir.—Enclosed, you will find a transcript of notes taken by me, in the case of John Shea. You will, no doubt, prefer giving a history of the accident, and the manner of removing the fractured bone. I will, therefore, confine myself to his subsequent condition from day to day.

June 26th—the day after the accident, found my patient very comfortable; ascertained that he had passed an easy night, and had some refreshing sleep. Pulse 84; tongue slightly furred; skin natural; had no delirium. Sulph. Magnesiæ ℥ i.

June 27th—did not pass so good a night as the preceding; pulse 83, skin hot. Sulph. Magnesiæ had operated well, the wound discharging a little sanious fluid with portions of lacerated brain.

June 28th—complains of pain in the head, some degree of thirst, with more heat of skin; pulse 32. Took 20 oz. blood from the arm, which relieved the pain in the head; wound discharging dark colored blood, and small pieces of brain.

June 29th—seems relieved of the unpleasant sensation about the head; pulse 78, skin natural and moist; bowels moved in the early part of the day; appetite very good.

June 30th—improving; no febrile disturbance, slept well; pulse 75.

July 1st—in all respects better; little or no pain in the head; tongue cleaning.

July 2d—had a comfortable night; wound discharging healthy pus, and in all respects improved since yesterday.

July 3d—in all respects improving; patient sat up a short time in bed; appetite good; wound granulating.

July 6th—has improved since last visit; granulations healthy and natural, wound healing; removed the poultices which have been continued as a dressing since the first; applied a pledget of lint and ung. zinci.

July 10th—found patient in all respects improving; has been sitting up half an hour each day since my last visit; wound looking well, appetite good; has had some meat broth and rice.

July 17th—continues to improve.

July 19th—sent for in haste, and being out of town, Dr. Pratt saw the patient; ascertained he had been down stairs and walked out; had eaten the day before a hearty meal of boiled pork and potatoes. Dr. P. bled him 15 oz. and ordered salts and senna.

July 20th—found him delirious, with much febrile excitement; had had chills through the night; pulse 108, skin hot and dry. Bled 15 oz. and ordered an injection to move the bowels. I was informed that he went to ride on the 18th and 19th, and pumped water. The new skin covering the exterior surface of the wound was protruding as if it contained fluid. Remained with my patient four hours, and after many pleasing prospects of recovery but a few days before, I now found him rapidly sinking. He lived until the next day at 3 P. M. Could not procure a post mortem examination.

Yours &c.,

F. L. HARRIS.

Remarks by the Editor.—The preceding case illustrates the fact that extensive injury of the brain and its membranes, and loss of cerebral substance, in cases of fracture, do not necessarily involve a great degree of local inflammation, general disorder, nor much apparent mental disturbance. The history of this case, would also seem to illustrate the rapidity of restoration, since, from the symptoms for 21 days after the accident, it is to be inferred that the patient was in a fair way to arrive at a speedy and complete recovery. Premature exertion, and other imprudences, which so often thwart the kindly efforts of nature, and the care of the Physician, appear to have occasioned the sudden change in the aspect of the case, and i's fatal termination.

